# Learning through “Research Cognitive Theory”: A new framework for developing 21st century research skills in secondary school students

**DOI:** 10.1016/j.heliyon.2025.e41950

**Published:** 2025-01-15

**Authors:** Noora J. Al-Thani, Zubair Ahmad

**Affiliations:** Qatar University Young Scientists Center (QUYSC), Qatar University, 2713, Doha, Qatar

**Keywords:** High-impact practices, Capacity building, Secondary education, Evidence-based instructional practice, Research experience

## Abstract

This study introduces a novel framework for High School Research Cognitive Learning Program (HSRCLP), focused on developing 21st century research skills (including research behavior, scientific identity, and values for the scientific community) among high school students. Drawing on a novel framework of “Research Cognitive Theory” (RCT) and empirical evidence, the HSRCLP represents a paradigm shift in secondary education, offering a dynamic research learning environment to enculturate research skills in students. The RCT in the HSRCLP is grounded within Social Learning Theory (SLT) & Social Cognitive Theory (SCT), incorporating the principles of challenge-based learning (CBL) and blended problem & project-based learning (PBL + PjBL) pedagogical approaches. To demonstrate its effectiveness, a two-group pre-post survey method was employed, involving 230 high school students across 10 batches. Statistical analysis revealed significant improvements in research attitudes among high-performing students (experiment group 1) exposed to the HSRCLP compared to the control group. Subsequent intervention for average or low-performing students (experiment group 2) also yielded substantial gains in research attitudes, highlighting the program's effectiveness across academic spectrums. Therefore, the HSRCLP demonstrates promise in bridging the gap between theory and practice, offering secondary education practitioners an evidence-based approach to nurture secondary students' 21st research century skills. Educators and policymakers are encouraged to integrate RCT-based frameworks into curricula and explore its scalability in various educational settings to enhance its impact on developing scientific identity and research-driven behaviors in students.

## Highlights

• Introduces High School Research Cognitive Learning Program (HSRCLP).
• HSRCLP focuses on nurturing research behavior, identity, and scientific values.
• Offers secondary educators an evidence-based approach to teaching research skills.

## 1 Introduction

In an increasingly complex world, the role of education in shaping the future workforce cannot be overstated. As industries across the globe continue to evolve, there is an ever-growing demand for individuals who can think critically, solve problems creatively, and contribute to scientific and technological innovations [1]. Central to meeting this demand is the need for high-quality STEM (Science, Technology, Engineering, and Mathematics) education that equips students with not just technical knowledge, but also the cognitive and interpersonal skills required to thrive in a rapidly changing global environment [2]. The development of these competencies is not only vital for individual student success but is also crucial for the continued advancement of scientific and technological fields that drive economic and societal progress.

However, traditional educational models have struggled to provide the deep, research-driven inquiry necessary for fostering the higher-order thinking skills required in STEM disciplines [3]. While STEM education typically emphasizes the acquisition of subject-specific knowledge, there is a growing recognition that fostering innovation and critical thinking requires more than just the ability to recall facts or apply formulas [4]; [5]. In fact, these skills are best developed through hands-on research, experimentation, and problem-solving—activities that mirror the actual processes used by scientists and innovators in real-world settings [6].

### 1.1 Research experiences in high school

Research Experience Programs (REPs) have been identified as one way to engage students in meaningful, real-world learning [7]. These programs provide students with opportunities to participate in scientific research, where they not only learn about research methodologies but also apply them in practical contexts, which improves their understanding of scientific knowledge [8]. These programs develop research abilities of students by encouraging critical thinking, problem-solving, analysis, and dissemination. REPs have traditionally been implemented primarily at the university level; however, in recent decades, there has been a noticeable shift toward implementing REPs in secondary and elementary schools [9]; [10]. High school, in particular, is an ideal time to engage students in REPs, creating a better understanding of subject matter and engaging them in collaborative and independent research projects. For this, High School Research Experience Programs (HSREPs) provide the platform to develop high school students’ intellectual and professional growth through concept understanding and scientific practice. As a result, students engage in an exploration process based on their interests and gain exposure to potential careers in research-oriented sectors [11]. Furthermore, pre-college research experiences help students develop research self-efficacy, which increases their motivation and confidence in performing research during their college years [12,13].

When students are exposed to research experiences, they get an awareness of the inquiry process, sharpen their problem-solving abilities, familiarize data collection procedures, and gain competency in deducing important research conclusions. The inquiry process represents the actions, conceptual needs, and values of “authentic science” [14]. However, it is important to note that Research Experience Programs (REPs) lack worldwide uniformity, with research revealing differences between approaches [15]; [16]. For example, while inquiry-based education frequently includes more “hands-on” components, it may not always prioritize being “minds-on.” The lack of clearly stated aims in the inquiry process might undermine the authenticity of a research experience (RE). Simultaneously, a reliance on high-stakes standardized examinations has diverted attention away from lab-based investigations. As a result, efforts have been made to incorporate actual research methodologies into secondary education, with the goal of engaging students in effective knowledge-based learning [17,18]. Significant efforts include Australian teachers creating specific chemistry contexts to give students more independence and extended time for experiments. In Germany, pre-experiment activities help students choose and design their own research projects. Additionally, the national curriculum in the United Kingdom emphasizes research exploration in school science subjects.

### 1.2 Challenges in existing HSREPs

Over the past several decades, efforts to implement REPs in secondary education have been met with mixed results. While some programs have proven successful in promoting scientific inquiry and developing research competencies among students, there are still significant gaps in how these programs are designed and evaluated [6]. A key challenge is the lack of a cohesive, evidence-based framework that can provide consistent guidance on how to structure these experiences in a way that maximizes student engagement and learning outcomes [19,20]. This inconsistency has led to challenges in ensuring that REPs provide the deep, research-driven inquiry that is essential for fostering the cognitive and behavioral growth of students. While efforts are being carried out by educators to provide research experiences for high school students, there is a lack of research in developing a replicable learning framework that is theoretically grounded and empirically established for imparting research experiences in schools [[19], [20], [21]]. This is important because navigating the research process independently poses several challenges, from balancing the focus on both the final product and the learning process to developing essential skills and overcoming common obstacles [22]. A key factor in this journey is the role of a learning framework, which is instrumental in providing the pathway to achieve this. Furthermore, high school educators, while knowledgeable in their fields, often lack experience working with high school students, which can limit their effectiveness as mentors (Lescak et al., 2019). Also, high school students, with their limited scientific background and more restrictive schedules compared to undergraduates, require educators to mentor them through a robust learning framework which can accommodate these constraints. To address these challenges and maximize the benefits of research experiences, it is essential to develop a theoretically grounded learning framework to improve instructional practices in both schools and research institutions.

### 1.3 Research cognitive theory (RCT)

At the heart of this issue lies the need for a pedagogical model that effectively bridges the gap between theory and practice—one that integrates research into the learning process in a way that is grounded in cognitive development theories. HSREPs require a research-rich learning framework to foster intrinsic motivation, self-efficacy, and intellectual growth. It is crucial to engage high school students in authentic learning processes, not just through knowledge acquisition but also a deep engagement with the scientific method. Therefore, the theoretical basis for this study is rooted within Research Cognitive Theory (RCT) which postulates intellectual learning occurs in a dynamic research environment and as a result of reciprocal interaction of the individual, environment, and behavior (Book on Research Learning Theory, under publishing with Springer). The distinctive feature of the RCT is the consequence of dynamic research environment's influence and its emphasis on intrinsic intellectual reinforcement. The dynamic research experiences influence reinforcements, prospects, and beliefs, all of which shape a person's engagement in intellectual learning. Therefore, RCT describes the influence of individual research practices on the development of intrinsic motivation, and influential factors on individual intellectual behaviors. Also, the theory provides opportunities for intellectual support through instilling expectations, self-efficacy, and using research learning and other reinforcements to achieve behavior change. Hence, in summary, the RCT points out the role of research activities in shaping intellectual behavior in contrast to Social Cognitive Theory (SCT), which emphasizes the role of mental processes in shaping behavior [23], and Social Learning Theory (SLT), which focuses on the role of observation and imitation [24].

Therefore. this study presents a novel teaching and learning framework for High School Research Cognitive Learning Program (HSRCLP) based on RCT, aimed at nurturing 21st century skills in high school students. Moreover, the study will empirically establish the effectiveness of this program over traditional HSREPs. By moving away from traditional HSREPs and towards a more conceptually rooted, deliberate, and outcome-oriented approach, this integration has the potential to significantly improve the effectiveness and impact of HSREPs.

In summary, the study aims to answer the following research question.

(1) To what extent does the High School Research Cognitive Learning Program (HSRCLP) enhance student's research attitudes?

## 2 Method

### 2.1 Design and development of the High School Research Cognitive Learning Program (HSRCLP)

The HSRCLP commenced with a call for research proposals, inviting participation from research faculties of the university's research center. Research proposals were reviewed by program experts based on their content, importance, alignment with the call for proposal. Subsequently, participating students were cordially invited to attend an orientation session, where they were presented with the diverse array of research projects and their respective faculty mentors. This pivotal session served not only to facilitate the project selection for students based on their interests, but also fostered meaningful connections between students and mentors. This helped to nurture a collaborative research environment conducive to academic growth and scholarly advancement.

**Fig. 1:**
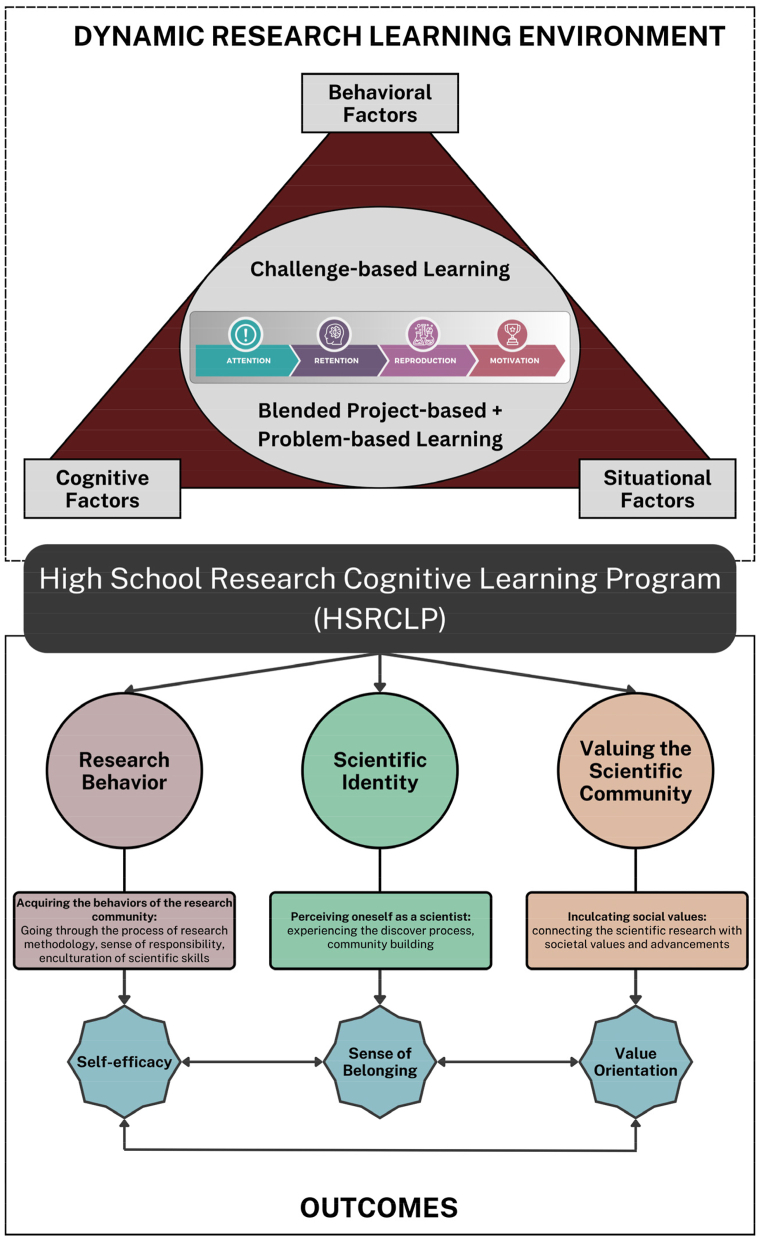
Framework for the High School Research Cognitive Learning Program (HSRCLP). Figure by authors.

Further, the HSRCLP was designed to provide students with a dynamic research learning environment (see Fig. 1) using the theoretical underpinnings of the Social Learning Theory (SLT) [24] and Social Cognitive Theory (SCT) [23]. The learning process is instigated using the steps: attention, retention, reproduction, and motivation. Learning activities were carefully designed to capture the students’ attention, ensuring they are cognitively engaged with the material at hand. Retention was subsequently sustained through brainstorming activities that encourage students to delve deeper into the topic and forge meaningful connections with the learned concepts. Further, students are provided with hands-on, experimental activities to reproduce the learned knowledge and solidify their understanding through active practice. Finally, the program takes advantage of positive reinforcements to sustain motivation throughout the learning journey. These reinforcements include prizes, competitions, and even recognition at the national level for the students.

This learning process at each of its step (attention, retention, motivation, reproduction) is delivered to the students using the pedagogical approach of challenge-based learning (CBL) [25] and blended problem-based and project-based learning (PBL + PjBL) [26]. CBL provides the foundation with real-world challenges designed just beyond the knowledge and caliber of the student inducing the learner to explore all the possible solutions. This impels the students to go one step ahead in their thinking, i.e., towards out-of-box thinking. Further, PBL and PjBL then build upon this by introducing a driving question that guides student research and inquiry. Finally, PjBL's emphasis on hands-on application allows students to solidify their understanding by reproducing the learned concepts through experiments. This integration of the learning process with the selected pedagogical approaches provides a dynamic research learning environment for the students.

It should be noted here that SLT plays a crucial role in the program by fostering observational learning, where students observe and imitate the research behaviors of mentors and peers, helping them internalize research skills and develop a scientific identity. Through peer-to-peer collaboration and mentorship, students learn by modeling the actions of those around them, reinforcing SLT's principles of learning through observation and interaction. In parallel, SCT's focus on self-efficacy, self-regulation, and reciprocal determinism is reflected in the program's structure. The program actively nurtures research self-efficacy by encouraging students to engage in hands-on research tasks, providing feedback from mentors, and offering a supportive environment. This enhances students' belief in their capacity to succeed in scientific inquiry, which aligns with SCT's emphasis on personal agency and cognitive development. The program's steps of attention, retention, reproduction, and motivation are directly tied to SCT's framework, as they engage students in cognitive tasks that foster deeper learning and self-efficacy. By applying both theories, the program effectively enhances research attitudes, scientific identity, and cognitive skills, aligning with the overarching objective of fostering 21st-century research competencies. This dynamic integration of SLT and SCT supports the program's goals of improving research attitudes and behaviors, ensuring that students experience both cognitive growth and behavioral change through an interactive and supportive research environment.

This dynamic research learning environment demonstrates the triadic reciprocal causation of SCT, through the interplay of behavioral, cognitive, and environmental factors. Within the HSRCLP, behavioral factors of the students are manifested through directly engaging and practicing in the research tasks, observing successful outcomes which help to enhance their 21st century skills and self-efficacy. Secondly, their cognitive (or personal) factors are explained by their attitudes, beliefs and motivation when being exposed to the dynamic research learning environment. Lastly, the environment is deliberately fostered to be interactive and supportive, where students engage with peers, mentors, and research projects explain the influence of environmental factors.

### 2.2 Study participants

The program included a total of 230 high school students, comprising 122 females and 108 males from 71 schools, encompassing both public and private institutions nationwide. Random sampling was used to ensure that a representative sample of high school students from various schools and performance levels was included. This was done to minimize selection bias and ensured the diversity of students in both experimental and control groups. These 230 participants engaged in research over a decade, spanning from 2010 to 2020, across 10 batches and contributing to 110 projects. Amongst the total students, 194 participants are considered as the experiment group (exposed to HSRCLP) and 36 students as the control group. Further, among the 194 students from the experiment group, 65 students were high performers (experiment group 1) and 129 were average or low performers (experiment group 2). This distinction based on performance was provided by the respective school teachers based on the students' grades. To minimize potential bias, care was taken to match students in the control group with similar academic performance levels to those in the experimental groups. First, the HSRCLP intervention is executed for high performers and compared with a control group. This establishes a baseline of performance and helps to understand the effectiveness of the HSRCLP under optimal conditions, i.e., when used by students who are already performing well academically. Then, the HSRCLP is finally executed for average or low performing students to comprehensively evaluate the program's impact across the overall spectrum of academic performance. Moreover, the program was intervened for the students in small groups (batches) to facilitate personalized attention, foster peer collaboration, and optimize the effectiveness of mentorship and guidance.

### 2.3 Survey design

To assess the effectiveness of the program, the study employed a two group pretest-posttest survey method. First, experiment group 1 was subjected to the HSRCLP along with the control group subjected to a traditional cookbook laboratory program. Following the successful results of this intervention, experiment group 2 (student with average or low performance) were subjected to the HSRCLP.

The pre-post questionnaires for students (see Table 1) were designed to assess their student's research attitudes in the form of research behavior, scientific identity, and values towards the scientific community as theorized by the Tripartite Integration Model of Social Influence (TIMSI) [27]. This model provides the framework for elucidating how individuals' knowledge and exposure to research practices can lead to the adoption of behavioral norms typical among scientists and researchers. This model draws on Kelman's model on social influence, delineating key components such as the influencing agent and the target of influence [28]; [29,30].

**Table 1:** Factors and their respective indicators that are considered for T-test analysis.

Factors	Indicators	Cronbach Alpha
Research Behavior	I am interested in performing scientific research which is important for my future success	0.833
	I can easily identify the scientific problems in research	
	I am confident that I can solve scientific problems in research	
	It is important to think critically to solve research problems	
Valuing the Scientific Community	Scientific research helps to make the world a better place	0.701
	I value the opportunity to meet scientists who work on my research topic	
	Participating in scientific research helps me appreciate the knowledge and perspectives of the scientific community	
Scientific Identity	I see myself as someone who can easily identify and address scientific problems	0.678
	I would like to join a scientific specialty at the university	
	I would like to have a career as a scientist or a researcher	

### 2.4 Data collection and analysis

The questionnaires were administered to students before and immediately after the program, employing pen and paper method. All participants gave their informed consent in written format for inclusion before they participated in the study. The ordinal variables within the questionnaires measured participants' agreement with statements using a one-dimensional psychometric response five-point Likert scale (ranging from highly disagree to highly agree). The 10 measures used to assess participants' research attitudes were combined to create quantitative construct to answer the study's research questions. Survey responses from students were measured by giving numerical values (strongly disagree = 1; disagree = 2; neutral = 3; agree = 4; strongly agree = 5), which were then added together to form a score known as the Research Attitude Score (RAS).

Further, Cronbach's alpha (α) was employed to evaluate the internal consistency of reliability. The calculated α values for each survey construct are presented in Table 1. As indicated by researchers [31], alpha values above 0.60 are considered reliable. The estimated alpha coefficients in this study demonstrated a reliable to highly reliable scale. To determine statistical difference, normality tests were conducted using the Shapiro–Wilk test to check the data distribution to choose between parametric and non-parametric significance tests. The data was found to be normally distributed and therefore we used paired sample T-tests using IBM SPSS Statistics version 27.0. Probability values that fell below the specified alpha threshold of 0.05 were considered statistically significant.

## 3 Results

Table 2 presents the results of significance tests comparing the research attitudes of the experimental group 1 (high performers) and the control group, using paired T-tests to evaluate pre- and post-RAS data. As shown in Table 2, the control group demonstrated only a negligible increase in mean scores with no statistical significance. In contrast, the experimental group 1 exhibited a substantial increase in mean scores, which was statistically significant. Specifically, for the control group, the difference in means between the pre-test (mean = 3.87) and post-test (mean = 3.96) was minimal and not significant (P = 0.677). However, in experimental group 1, there was a notable improvement in research attitudes, with mean scores rising from 3.68 in the pre-test to 4.26 in the post-test, and the difference was statistically significant (P < 0.001). This highlights the effectiveness of the intervention in enhancing research attitudes among high-performing students.

**Table 2:** Comparison of the paired T-test results for experiment group 1 and the control group.

Experiment Group 1					Control Group				
Batch	N	*Pretest* Mean (SD)	*Posttest* Mean (SD)	p	Batch	N	*Pretest* Mean (SD)	*Posttest* Mean (SD)	p
B1	36	3.78 (0.573)	4.17 (0.572)	0.013	B2	14	3.64 (1.499)	3.79 (1.017)	0.689
B3	29	3.56 (0.324)	4.39 (0.550)	0.001	B4	22	4.03 (0.429)	4.07 (0.466)	0.454
Total	**65**	**3.68 (0.487)**	**4.26 (0.568)**	**0.000**	**Total**	**36**	**3.87 (0.991)**	**3.96 (0.731)**	**0.677**

Table 3 presents the results of paired T-tests used to evaluate the research attitudes of average and low-performing students (experiment group 2) before and after the HSRCLP intervention. As shown in Table 3, there was a considerable increase in mean scores for all batches from pre-to post-test, with the differences being statistically significant. The intervention in experiment group 2 led to a marked improvement in research attitudes, with mean scores rising from a pre-test mean of 1.97 to a post-test mean of 3.09. The difference was highly significant (p < 0.001), indicating the success of the HSRCLP intervention in enhancing research attitudes among average and low-performing students.

**Table 3:** Paired T-test results for the experiment group 2.

Experiment Group 2				
Batch	N	*Pretest* Mean (SD)	*Posttest* Mean (SD)	p
B5	24	2.22 (0.645)	2.84 (0.896)	0.019
B6	16	1.83 (1.211)	3.42 (0.614)	0.001
B7	23	1.65 (0.342)	3.20 (0.914)	0.001
B8	28	2.00 (0.682)	3.32 (0.814)	0.001
B9	22	1.94 (0.594)	2.69 (0.346)	0.001
B10	16	2.19 (0.980)	3.17 (1.010)	0.009
Total	**129**	**1.97 (0.757)**	**3.09 (0.823)**	**0.000**

## 4 Discussion

The current study's findings provide significant insights into the effectiveness of the High School Research Cognitive Learning Program (HSRCLP) in enhancing research attitudes among high school students. By incorporating research-driven pedagogical practices, the HSRCLP presents a promising framework for fostering key research behaviors, scientific identity, and values for the scientific community. This discussion section reflects on the study's findings and connects them to broader literature, emphasizing the program's relevance and potential contributions to educational research.

Specifically, results from the first experimental group, consisting of high-performing students, revealed a significant improvement in research attitudes following the intervention. The substantial increase in post-test scores, as compared to the control group, indicates that the HSRCLP positively impacted students' perception and engagement with research. This finding aligns with prior studies that emphasize the importance of research-based learning environments in fostering intellectual curiosity and engagement among students [14,32,33]. These results support the argument that high-performing students, when placed in an environment that integrates research-driven pedagogy, can experience significant growth in their research-related attitudes, a key indicator of success in future scientific endeavors [7].

For the second experimental group, consisting of average and low-performing students, the results demonstrate that the HSRCLP was equally effective in enhancing research attitudes. The statistically significant increase in mean scores of post-intervention highlights the program's potential to engage a broader spectrum of students, not just those who are high achievers. This is consistent with research that show that such interventions are effective for average and low-performing students [34,35]. The substantial gains observed among average and low-performing students underscore the inclusivity and flexibility of the HSRCLP, making it a valuable tool for improving research attitudes across varied academic performance levels.

As outlined in the TIMSI model, exposure to scientific knowledge and practice helps individuals integrate into the community of scientists and researchers. This integration is dependent on their acquisition of scientific knowledge, which improves efficacy, identity, and values as depicted in the Educational TIMSI model (E-TIMSI) [27] (see Fig. 1). Prior research has demonstrated that these integration indicators predict both short- and long-term engagement in the community's normative behaviors [30,36]. As a result, efficacy, identity, and values moderate the relationship between scientific knowledge and engagement in science-related activities. Therefore, in the present study, the dynamic research learning environment provided to the students in the HSRCLP proved to be effective in achieving the indicators of the TIMSI model.

Such a learning framework satisfies the calls for effective reform of highly integrated and supportive models for high school students that is required to aid them in selecting research career paths and igniting their enthusiasm for the subject [6]. Furthermore, in many countries, STEM-based HSREPs are integrated into classroom activities, promoted by educational authorities through school projects, contests, and seminars, and even incorporated into curricular courses. As a result, many of these experiences remain unreported, and there has been inadequate emphasis on standardizing these research experiences into formal programs at the regional or worldwide levels. Therefore, previous study by Zubair et al. [6] deemed it critical to perform more targeted investigations with specialized learning models that can support students’ scientific progress beyond traditional research programs. The teaching and learning framework provided by the HSRCLP provides the ground to standardize research experiences to go beyond traditional research programs.

It is also important to note that the current study's findings not only provide insights into comparing the traditional research programs with the novel HSRCLP framework, but they also identify the important aspects of student outcomes (as depicted in Fig. 1) that can be achieved through the HSRCLP's implementation. Firstly, cultivating a research behavior in students to induce the behavior of the research community in them, which develops self-efficacy. This self-efficacy according to Bandura [37], is an important social psychological predictor of individual participation in behaviors. It is defined as the belief in one's capabilities to organize and execute courses of action required to produce given attainments, and has been extensively studied for its tremendous impact on behavior [37]. According to SCT, student's conceptions of self-efficacy influences many parts of their lives, including their objectives, decision-making processes, effort levels, task selection, resilience in the face of adversity, and mental well-being [38]. Moreover, previous studies have demonstrated that individuals with high self-efficacy put forth more effort and persistence in their academic pursuits [39]. They display an ongoing interest in their studies and actively seek effective solutions for overcoming obstacles.

The second outcome is the enculturation of scientific identity in students, which according to the TIMSI model happens when people accept influence from others or groups in order to form or sustain a satisfactory self-defining relationship with them [30]. Here, acceptance is more likely when people share a social identity or self-concept with the relevant group, which leads to the development of a sense of belonging [40]. Studies show that when students develop this scientific identity and perceive themselves as scientists, it is associated with improved academic performance, increased retention and persistence in a science career [41]; [42,43]. Lastly, students develop values for the scientific community which is described as the concept of internalization, wherein people accept influence from others in order to fit their actions and beliefs with their own value systems [30]. In essence, when students are integrated into the research community, they comply with communal norms because they align with their own internalized values, which are shared by the greater community. Thus, leading to value orientation where values are anticipated to play an important role in predicting engagement in the student's activities.

In summary, the discussion of the study's findings not only aligns with existing literature on research-driven education but also underscores the unique contributions of the HSRCLP to fostering research attitudes across diverse student performance levels. By connecting these results to established educational theories and frameworks, this study highlights the broader relevance of the program and its potential to contribute significantly to educational research and practice.

## 5 Limitations

While this study offers useful insights, its reliance on quantitative data and small sample size necessitates further research. Future longitudinal research using mixed approaches can provide a more in-depth knowledge of the HSRCLP's effectiveness, revealing insight on its long-term impact on student performance. Incorporating qualitative methods, such as interviews or focus groups, could offer richer, more comprehensive insights into how students engage with the HSRCLP and how the program impacts their development over time. Secondly, the relatively small sample size restricts the generalizability of the findings. Larger, more diverse sample sizes are necessary to validate the program's effectiveness across different educational settings and student demographics. This limitation suggests the need for future studies to include a broader range of participants from various regions and socioeconomic backgrounds. Third, there were methodological limitations in controlling external variables, such as differences in mentor involvement, school resources, and varying levels of student preparedness. These factors may have influenced the outcomes, making it difficult to isolate the effect of the HSRCLP intervention. Additionally, contextual limitations must be noted. The study was conducted within a specific educational framework and cultural context, which may not fully translate to other regions or schooling systems. Variations in educational policies, school environments, and support systems could influence the replicability and success of the program in other contexts. Also, by transcending geographical boundaries and fostering collaboration on a global scale, these approaches have the potential to drive productivity and prosperity in the science fields, ultimately shaping a brighter future for generations to come.

## 6 Conclusions and future directions

High School Research Experience Programs (HSREPs) offer valuable opportunities for secondary students to engage in scientific inquiry. However, traditional approaches lack effectiveness in fostering essential research attitudes. Moreover, there is a dearth of studies which present a standardized theoretically grounded teaching and learning framework for HSREPs. Therefore, this study presents the HSRCLP with a novel teaching and learning framework by integrating theory with practice to cultivate a dynamic research learning environment for high school students. By adopting a multifaceted approach theoretically rooted in SLT & SCT and pedagogically grounded within CBL & PBL + PjBL, the findings of the study show that the program fosters the development of essential research attitudes in high school students. Moreover, the proposed intervention demonstrates effectiveness over traditional research programs in terms of research behavior, scientific identity, and value for the scientific community. These outcomes represent the enculturation of 21st century skills in the students.

Therefore, this study contributes to both educational research and practice by offering a concrete, scalable framework that bridges the gap between theory and practice in secondary education research programs. It provides a replicable model for fostering research attitudes, critical thinking, and engagement in STEM fields, helping educators prepare students for future scientific endeavors. Future research should focus on conducting longitudinal studies to assess the long-term impact of the HSRCLP on student performance, scientific engagement, and career trajectories. Additionally, incorporating mixed-method approaches, including qualitative data, can offer deeper insights into student experiences and the impact of mentor-student interactions. Educational policymakers should consider integrating research-driven frameworks like the HSRCLP into national curricula, particularly for STEM education. Providing funding and resources to scale such programs across diverse educational settings can promote widespread adoption and support the development of the next generation of innovators and researchers.

## CRediT authorship contribution statement

**Noora J. Al-Thani:** Supervision, Conceptualization. **Zubair Ahmad:** Writing – original draft, Investigation, Formal analysis, Data curation.

## Ethics statement

This study was reviewed and approved by Qatar University Institutional Review Board (QU-IRB) with the approval number 2187481-1.

## Funding

This work was supported by Qatar University Grant # QUT2RP-YSC-24/25-536. The findings achieved herein are solely the responsibility of the authors.

## Data availability

Data will be made available on request.

## Declaration of competing interest

The authors declare that they have no known competing financial interests or personal relationships that could have appeared to influence the work reported in this paper.
